# Integrated metabolome and transcriptome analyses reveal the molecular mechanism underlying dynamic metabolic processes during taproot development of *Panax notoginseng*

**DOI:** 10.1186/s12870-024-04861-8

**Published:** 2024-03-05

**Authors:** Xuejiao Li, Yan Zhao, Shuilian He, Jing Meng, Yingchun Lu, Huineng Shi, Chunlan Liu, Bing Hao, Qingyan Tang, Shuangyan Zhang, Guanghui Zhang, Yu Luo, Shengchao Yang, Jianli Yang, Wei Fan

**Affiliations:** 1 State Key Laboratory of Conservation and Utilization of Bio-Resources in Yunnan, The Key Laboratory of Medicinal Plant Biology of Yunnan Province, National and Local Joint Engineering Research Center on Germplasm Innovation and Utilization of Chinese Medicinal Materials in Southwest China, Yunnan Agricultural University, Kunming, China; 2 College of Landscape and Horticulture, Yunnan Agricultural University, Kunming, 650201 China; 3 College of Food Science and Technology, Yunnan Agricultural University, Kunming, 650201 China

**Keywords:** *Panax notoginseng*, Taproot development, Metabolic changes, Gene expression, Triterpenoid saponins

## Abstract

**Background:**

*Panax notoginseng* (Burk) F. H. Chen is one of the most famous Chinese traditional medicinal plants. The taproot is the main organ producing triterpenoid saponins, and its development is directly linked to the quality and yield of the harvested *P. notoginseng*. However, the mechanisms underlying the dynamic metabolic changes occurring during taproot development of *P. notoginseng* are unknown.

**Results:**

We carried out metabolomic and transcriptomic analyses to investigate metabolites and gene expression during the development of *P. notoginseng* taproots. The differentially accumulated metabolites included amino acids and derivatives, nucleotides and derivatives, and lipids in 1-year-old taproots, flavonoids and terpenoids in 2- and 3-year-old taproots, and phenolic acids in 3-year-old taproots. The differentially expressed genes (DEGs) are related to phenylpropanoid biosynthesis, metabolic pathway and biosynthesis of secondary metabolites at all three developmental stages. Integrative analysis revealed that the phenylpropanoid biosynthesis pathway was involved in not only the development of but also metabolic changes in *P. notoginseng* taproots. Moreover, significant accumulation of triterpenoid saponins in 2- and 3-year-old taproots was highly correlated with the up-regulated expression of *cytochrome P450s* and *uridine diphosphate-dependent glycosyltransferases* genes. Additionally, a gene encoding RNase-like major storage protein was identified to play a dual role in the development of *P. notoginseng* taproots and their triterpenoid saponins synthesis.

**Conclusions:**

These results elucidate the molecular mechanism underlying the accumulation of and change relationship between primary and secondary metabolites in *P. notoginseng* taproots, and provide a basis for the quality control and genetic improvement of *P. notoginseng*.

**Supplementary Information:**

The online version contains supplementary material available at 10.1186/s12870-024-04861-8.

## Background

Plant growth and development are regulated by both internal factors such as genetic make-up and hormone levels as well as external cues such as light, temperature, water, and nutrients. To adapt to the rapidly fluctuating environment and meet their own metabolic needs, the growth and development of plants require dynamic metabolic processes throughout the whole life cycle [1]. Primary metabolic processes such as glycolysis, the tricarboxylic acid cycle and starch synthesis provide energy for plant growth and storage substances for development [2–5]. For example, the accumulation of starch promotes the formation of tubers and roots, while carbohydrate metabolism is involved in regulating plant growth, especially cell division and expansion [6]. In contrast, plant secondary metabolic processes play an important role in regulating the response of plants to development and stress factors. These secondary metabolites not only contribute to the aroma, taste and color of flowers, and attract pollinators and seed dispersers, but may also act as defensive compounds or toxins [7–10]. However, the molecular mechanisms underlying the dynamic metabolic changes during plant growth and development are poorly understood.

The levels of accumulation of the active ingredients in medicinal plants determine their quality in Traditional Chinese Medicine. Most of these active ingredients are secondary metabolites, which are closely related to the developmental stages and growing seasons of medicinal plants [11–14]. In the commercial production of these plant products, the time when the total biomass and main medicinal ingredients are highest is often selected as the best harvest time. The contents of chlorogenic acid, rutin, and luteolin in *Lonicera japonica* are high at the slightly white alabastrum, whole white alabastrum and silver flower stages of flower development, respectively [12]. In *Rehmannia glutinosa*, the concentrations of catalpol are higher in young leaves, while levels of aucubin, an intermediate product of catalpol biosynthesis, gradually increase with the growth of the leaves [15]. Thus, the accumulation of and changes in medicinal plant metabolites are directly related to plant growth and development. However, the majority of studies in this field have focused on changes in active ingredients in different tissues and different growth stages from phenotypic level, and systematic studies regarding dynamic changes in metabolite composition during the growth and development of medicinal plants are lacking.

*Panax notoginseng* (Chinese ginseng, Araliaceae) is used in Traditional Chinese Medicine to treat hypertension, thrombosis, and atherosclerosis, and is also believed to have neuroprotective properties [16]. The taproot, which is abundant in triterpenoid saponins, is the main commercial organ, and its development directly determines the quality and yield of the crop. In cultivation, the plants are usually harvested in the third year of growth, when the triterpenoid saponins concentrations in the taproots are highest. Previous studies have found that concentrations of protopanaxadiol-(Rb1, Rc, Rb2, and Rd) and protopanaxatriol-type (R1, Rg1, and Re) saponins were higher in *P. notoginseng* taproots than in shoots, and the overall saponins content of 3-year-old roots was 1.4-fold higher than in 2-year-old plants [17, 18]. In addition, high accumulation of dammarene-type triterpene saponins in 3-year-old *P. notoginseng* taproots was associated with down-regulated expression of genes involved in primary metabolism and cell activity, and with up-regulated expression of genes related to secondary metabolism [19]. These results suggest that the synthesis and accumulation of triterpenoid saponins are closely related to the growth and development of *P. notoginseng*. However, the molecular mechanisms and substance basis underlying the dynamic processes of triterpene saponins accumulation in *P. notoginseng* are not clear.

In a previous study, we previously found that the molecular processes associated with plant hormone signal transduction, starch and sucrose metabolism, and phenylpropanoid biosynthesis triggered taproot thickening in 1-year-old *P. notoginseng* plants [19]. The dynamic changes in metabolites at different developmental stages are still to be fully described, but may be closely related to taproot development and saponins formation in *P. notoginseng*.

This study conducted integrated metabolome and transcriptome analyses with the aim of (1) further understanding the molecular mechanisms underlying the dynamic metabolic processes leading to *P. notoginseng* taproot thickening, and (2) investigating dynamic changes in metabolite composition and concentration, as well as gene expression in *P. notoginseng* taproots at different developmental stages (1-, 2- and 3-year-old). The results illustrate the dynamic changes occurring with time in *P. notoginseng* taproots, and the relationships between the primary and secondary metabolites present. The identification of potential genes provides a molecular basis for quality control and genetic improvement of future *P. notoginseng* crops.

## Results

### Growth of *P. notoginseng* taproots at different developmental stages

**Fig. 1:**
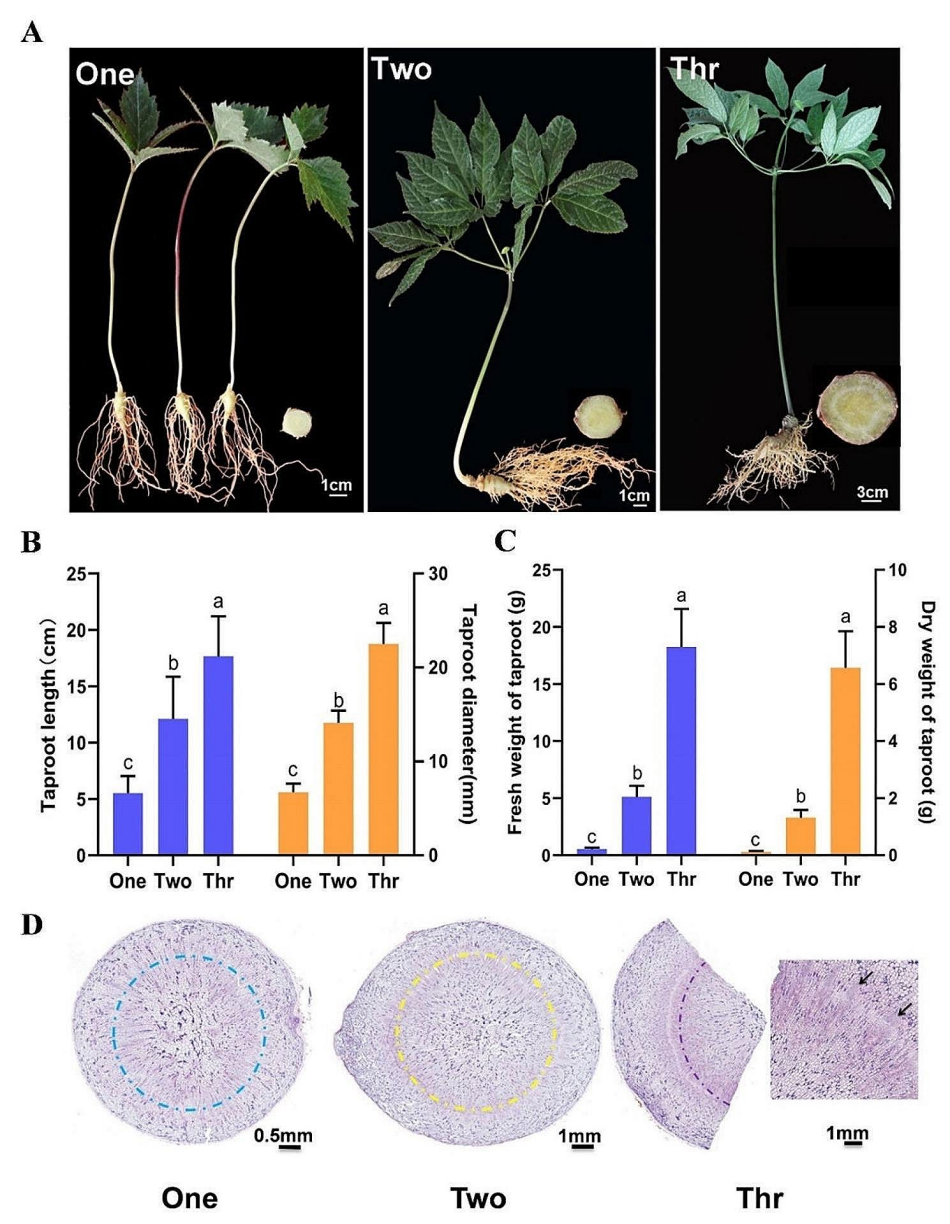
Morphology of *Panax notoginseng* taproots at different growth stages. Growth performance (**A**), taproot length and diameter (**B**), taproot weight (**C**), anatomical structure (**D**) of taproots in 1-, 2-, and 3-year-old plants. Dashed circles and arrows indicate the cambium and surrounding structures where cells divide significantly. Data are expressed as means ± SD (*n* = 30). Columns with different letters are significantly different at *P* < 0.05

To investigate the dynamic changes occurring during the development of *P. notoginseng*, the growth indexes, morphological structure and total saponins content of the taproots were measured over three growth years. The length, diameter, weight and total saponins content of the taproots were all found to significantly increase from 1-year-old to 3-year-old plants (Fig. 1B, C and Fig. S1), indicating that the levels of accumulated active ingredients are positively correlated with the year of growth years in *P. notoginseng*. Microscopic observation of the structure in paraffin section revealed that the showed significant cell division, especially around the cambium, over each of the three tested years (Fig. 1D). Whilst the xylem and phloem in 1-year-old taproots were significantly divided, a large number of xylem divisions were obvious for both 2- and 3-year-old taproots (Fig. 1D).

### Changes of metabolites in *P. notoginseng* taproots between different growth stages

**Fig. 2:**
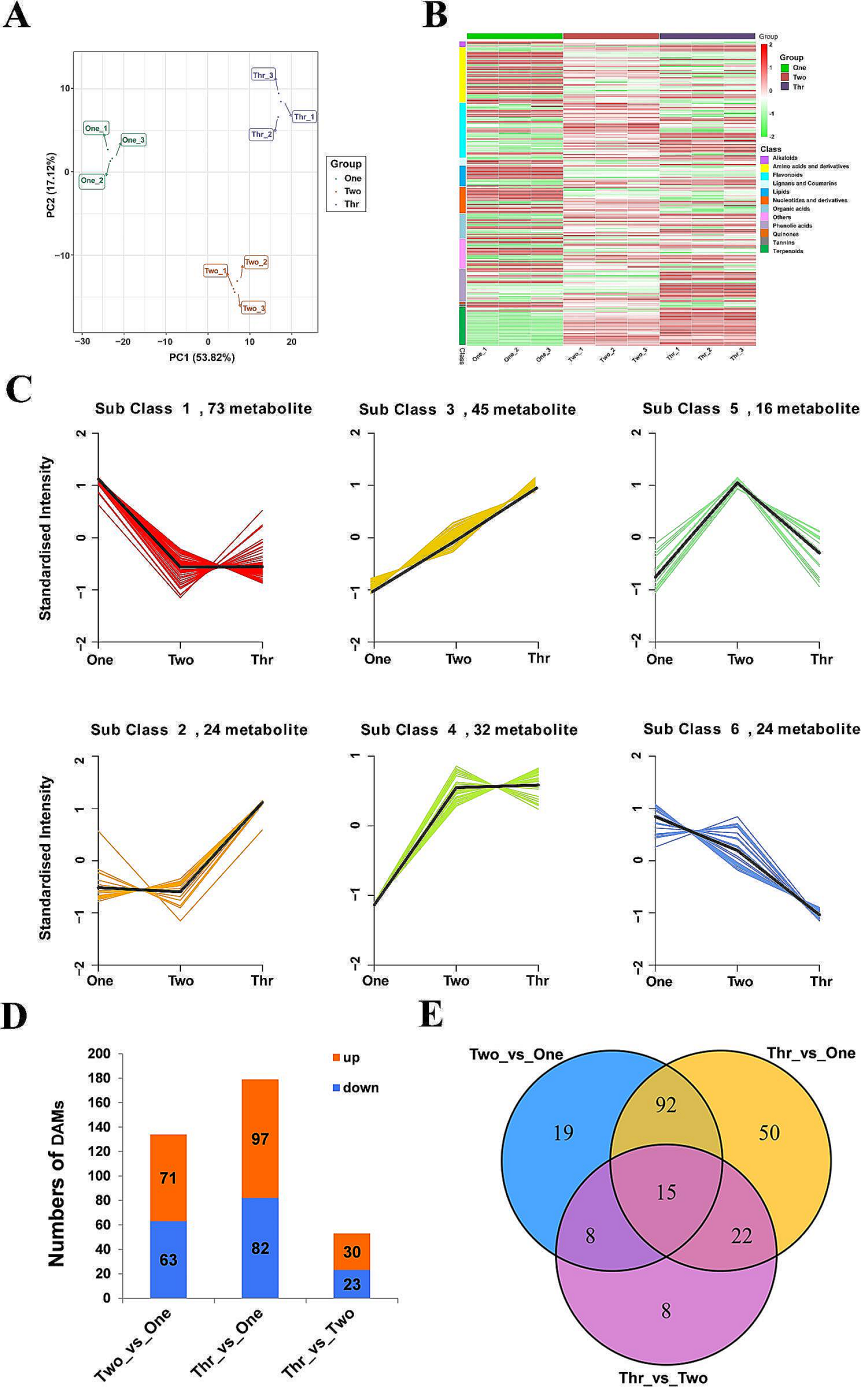
Multivariate statistical analysis of metabolome data in taproot samples at three different ages. (**A**) PCA score plot. (**B**) Cluster analysis of DAMs. The color indicates the relative concentration of metabolites from low (green) to high (red). (**C**) *k-*means clustering analysis of DAMs. (**D**) Numbers of metabolites with differences in relative abundance in different comparison groups. Orange and blue columns represent the numbers of metabolites with increases or decreases in relative abundance, respectively. (**E**) Venn diagram showing the overlapping and unique DAMs in comparison groups

To create a comprehensive landscape of metabolic change networks in taproots in 1-, 2- and 3-year-old plants, we performed a metabolic profiling analysis using a broad targeting LC − MS/MS-based method. A total of 445 distinct annotated metabolites, including flavonoids (19%), amino acids and derivatives (16%), nucleotides and derivatives (11%), lipids (10%), organic acids (10%), terpenoids (9%), phenolic acids (8%), saccharides and alcohols (6%), alkaloids (2%), lignans and coumarins (2%), and others (7%), were identified (Table S2 and Fig. S2A). Principal component analysis (PCA) and hierarchical clustering revealed that the three biological replicates from each group clustered tightly together, indicating that the metabolic data were highly repeatable and reliable (Fig. 2A and Fig. S2B).

To investigate the dynamic changes in the accumulation of the identified metabolites, orthogonal partial least-squares discriminant analysis (OPLS-DA) was performed, which produced 214 differentially accumulated metabolites (DAMs) in the 1-year-old to 3-year-old taproots (Table S3). Hierarchical clustering of DAMs showed that amino acids and derivatives, nucleotides and derivatives, and lipids accumulated significantly in the 1-year-old taproots, flavonoids and terpenoids were present at high levels in 2- and 3-year-old taproots, and phenolic acids accumulated predominantly in 3-year-old taproots (Fig. 2B). Interestingly, of the 28 differentially detected terpenoids, 26 terpenoids including 23 triterpenoid saponins, showed significant accumulation in the 2- and 3-year-old taproots, with the levels accumulated being generally higher in 3-year-old taproots than in 2-year-old taproots (Fig. S3), which confirms that the 3-year-old *P. notoginseng* has better harvest and medicinal value [14, 20]. Investigation of DAMs using *k-*means cluster analysis revealed 6 distinct clusters (Sub class 1–6), of which Sub class 1 contained the largest number of metabolites (Fig. 2C), reflecting the vigorous physiological activities of 1-year-old taproots. The DAMs in Sub class 1 had their highest levels only in 1-year-old taproots, and were mainly amino acids and derivatives, nucleotides and derivatives, and lipids. The DAMs in Sub class 5 were those with the highest levels only in the 2-year-old taproots, and these were mostly flavonoids. The DAMs in Sub class 2 were those with levels that were the highest only in 3-year-old taproots, and these chemicals were mostly phenolic acids. In comparison, Sub classes 3 and 4 contained DAMs with their highest levels across both 2- and 3-year-old taproots simultaneously, many of which were flavonoids and terpenoids. These results are consistent with the DAM hierarchical clustering analysis (Fig. 2B), indicating that the 1-year-old taproots mainly synthesize primary metabolites or precursors, while secondary metabolites accumulate predominently in 2- and 3-year-old taproots.

Meanwhile, DAMs from the three groups were subjected to a pairwise comparison according to their relative concentrations. These comparisons were divided into three categories, i.e. Two vs. One, Thr (Three) vs. One, and Thr vs. Two. The numbers of DAMs were 134 for Two vs. One, 179 for Thr vs. One, and 53 for Thr vs. Two (Fig. 2D), suggesting that there were significant differences in the metabolic profiles of taproots at different developmental stages, although the metabolic profiles of the 2- and 3-year-old taproots were relatively similar. This result was consistent with the results of the PCA analysis (Fig. 2A). By analyzing overlaps among three comparison groups (shown in the Venn diagram), 15 DAMs, common to all groups were identified, including amino acids and derivatives (2), organic acids (1), phenolic acids (3), alkaloids (2), terpenoids (5), and others (2) (Fig. 2E and Table S5). Of these, 4 triterpenoid saponins, including ginsenoside K, ginsenoside Rh2, ginsenoside Mc, and notoginsenoside K continued to accumulate in the taproots throughout the tested years (Table S5).

### Global expression profile analysis in *P. notoginseng* taproots at different growth stages

**Fig. 3:**
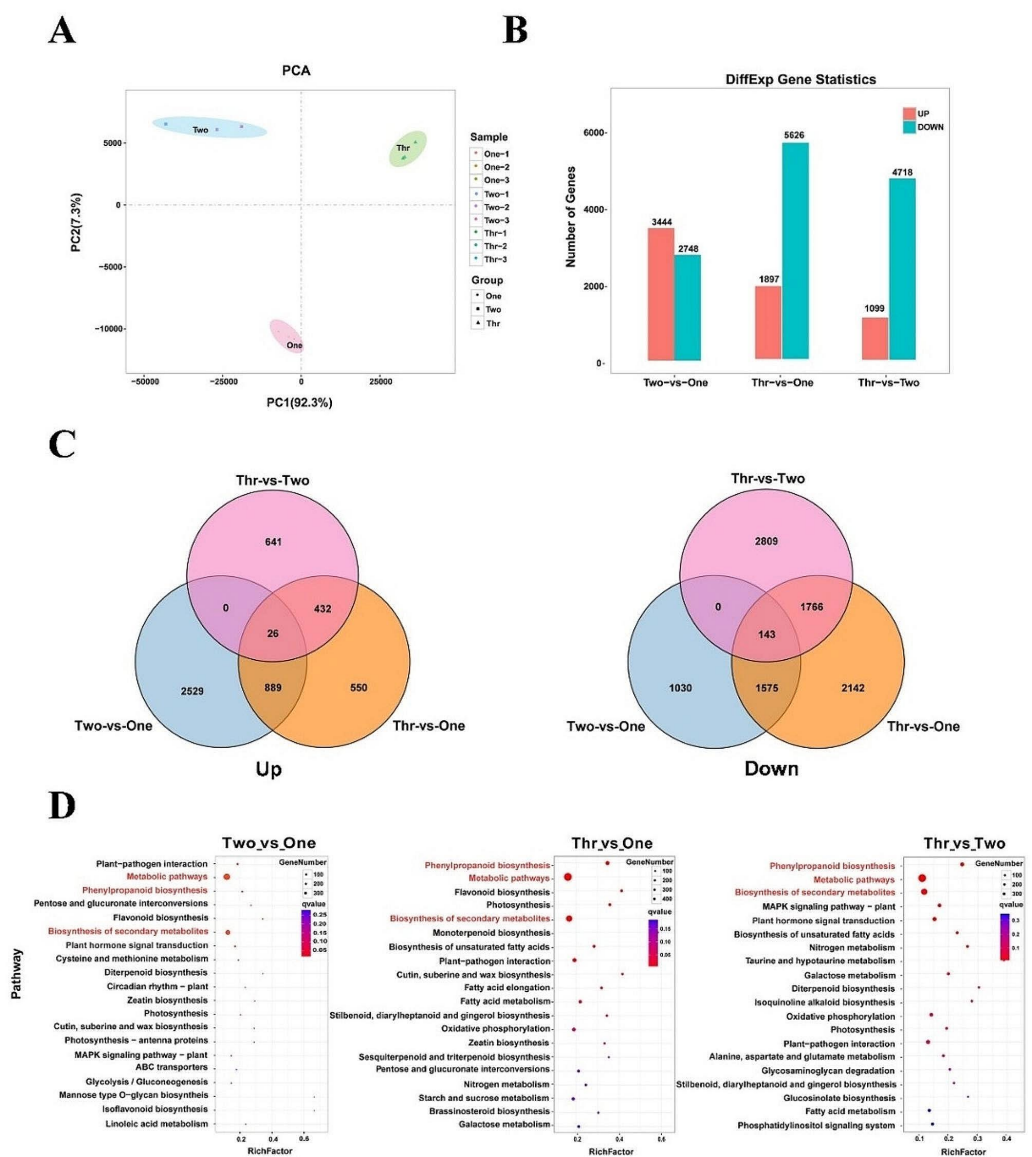
Multivariate statistical analysis of metabolome data in taproot samples of three different ages. (**A**) PCA score plot. (**B**) Numbers of genes with differences in relative abundance in different comparison groups. Red and blue columns represent the numbers of genes with increases or decreases in relative abundance, respectively. (**C**) Venn diagram showing the overlapping and unique DEGs in each comparison group. (**D**) KEGG pathways enrichment of the top 20 DEGs. The KEGG pathways, associated with the phenylpropanoid biosynthesis, metabolic pathway and biosynthesis of secondary metabolites are marked in red

To uncover the potential molecular basis of taproot development in *P. notoginseng*, we conducted global transcriptomic analyses using RNA-seq. A total of 33,099 unigenes were annotated by matching to the NCBI non-redundant protein sequences (NR), SWISS-PROT, eukaryotic ortholog groups (KOG), Kyoto encyclopedia of genes and genomes (KEGG) and gene ontology (GO) databases (Fig. S4). A principal component analysis (PCA) of the gene expression profiles showed that the major variance (PC1, contributing 92.3% variation) was able to separate the data according to the three different growth years. The second principal component (PC2, contributing 7.3% variation), separated the data between 1-year-old and 2- and 3-year-old samples. The 2 and 3-year-old were more similar to each other than either was to the 1-year-old samples (Fig. 3A). In order to obtain reliable gene expression profiles, reads with Log_2_ |Fold Change| > 1 and FDR < 0.05 were selected to annotate the differentially expressed genes (DEGs). A total of 6440 up-regulated DEGs and 13,092 down-regulated DEGs were derived from the pairwise comparisons (Fig. 3B; Table S6-S8). The number of down-regulated DEGs was more abundant than the number of up-regulated DEGs in all comparison groups except for the comparison Two vs. One (Fig. 3B). From the Venn diagram, 26 up-regulated DEGs and 143 down-regulated DEGs were found to be common to all comparison groups (Fig. 3C). Among the DEGs common to all groups, up-regulated DEGs were mainly related to signal transduction and transcription, stress and defense response, carbon metabolism, secondary metabolism and transport, while down-regulated DEGs were mainly involved in signal transduction and transcription, secondary metabolism, cell wall metabolism, stress and defense response and lipid metabolism (Table S9).

The DEGs were further categorized and characterized according to their functional category as defined by the GO and KEGG databases. GO analysis showed that the DEGs were mainly involved in “metabolic process”, followed by “cell process” and “single-organism process” in the “biological process” category. In the “cellular component” category, the DEGs were mostly involved in “cell, cell part and organelle”. In the “molecular function” category, “catalytic activity” was predominant, followed by “binding and transport activity” (Fig. S5). The enrichment analysis of KEGG indicated that the three metabolic pathways involved in “phenylpropanoid biosynthesis”, “metabolic pathway” and “biosynthesis of secondary metabolites” were predominantly enriched (Fig. 3D).

**Fig. 4:**
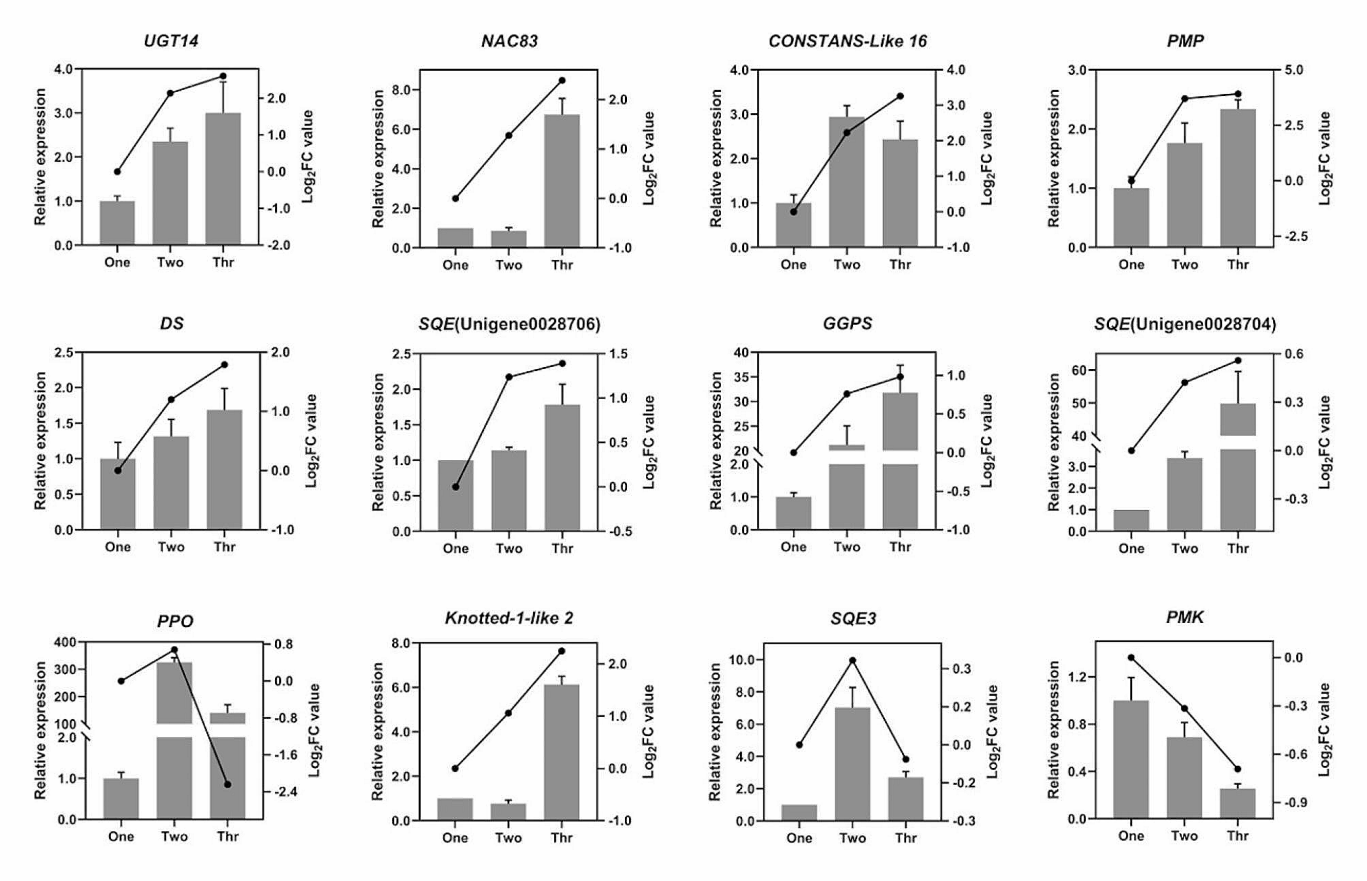
Expression patterns of candidate DEGs was assessed using RT-qPCR. Columns represent RT-qPCR results and lines represent RNA-Seq results. Left *Y*-axes indicate the relative mRNA expression levels of genes calculated using the 2^−ΔΔ*C*t^ method, while right *Y*-axes indicate the log_2_ fold changes from RPKM values calculated using 1-year-old taproots as a control. Data are means ± SD (*n* = 3)

To validate the reliability of the transcriptomic data, 12 DEGs were selected randomly for quantitative real-time PCR (RT-qPCR) analysis. There was a good correlation between the transcriptomic data and the RT-qPCR results (Fig. 4), suggesting that the transcriptomic data was able to accurately reflect the transcriptional changes during the development of *P. notoginseng* taproots.

### Differential expression of genes related to phenylpropanoid biosynthesis pathway

**Fig. 5:**
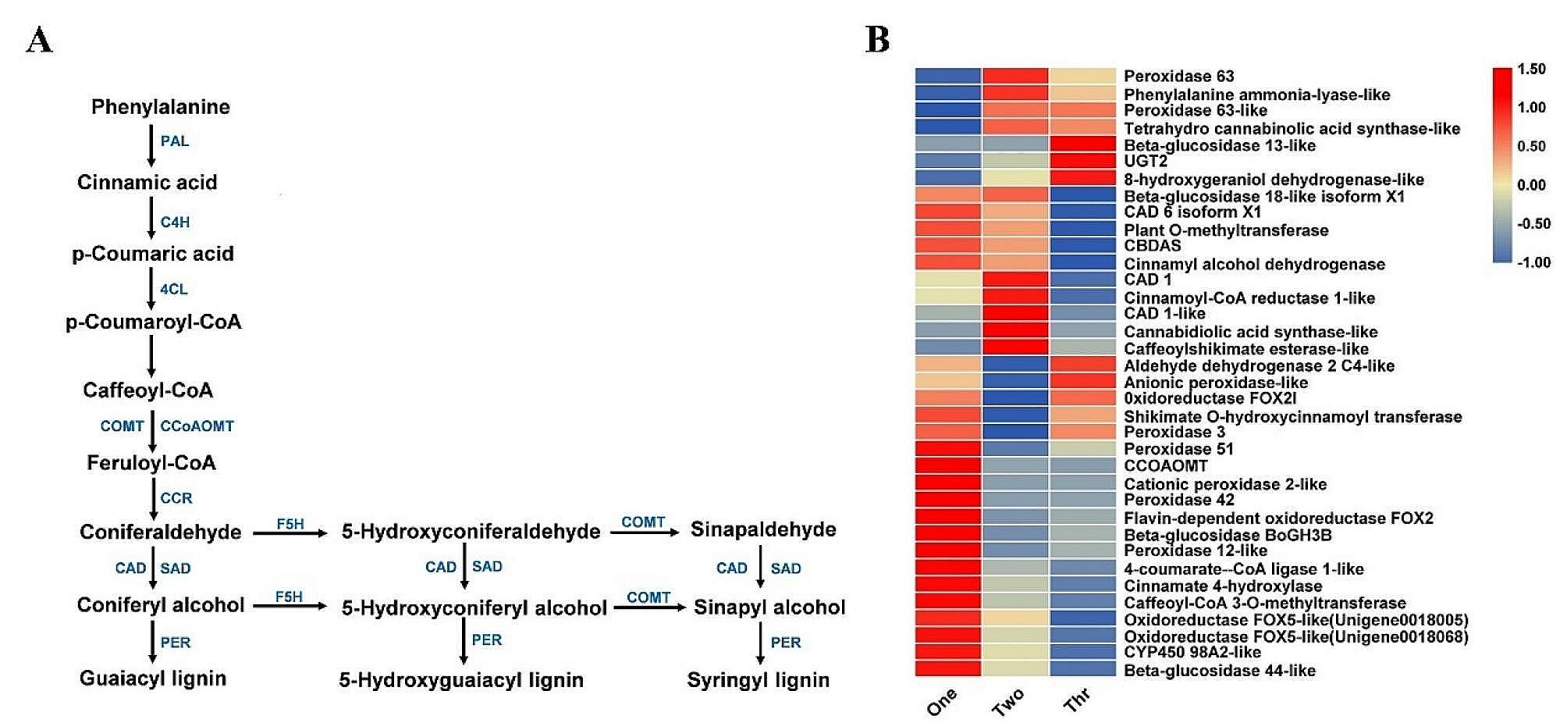
Significant enrichment pathway and DEGs related to phenylpropanoid metabolism. (**A**) Partial pathway of phenylpropanoid biosynthesis. (**B**) Heatmap of DEGs related to phenylpropanoid metabolism

The phenylpropanoid biosynthesis pathway was predominantly enriched in the taproots across all three studied years of growth, indicating that it plays an important role in the development and metabolism of *P. notoginseng* (Fig. 3D). Previous studies have reported that different branches of the phenylpropanoid biosynthesis pathway are involved in the synthesis of different products, including salicylic acid, flavonoids, lignin, coumarins, benzoic acid, proanthocyanidins, and others [21]. Further transcriptome analysis of the data generated in this project revealed that the DEGs were mainly concentrated on the biosynthetic pathway of lignin, including the biosynthetic pathway of guaiacyl lignin, 5-hydroxyguaiacyl lignin and syringal lignin (Fig. 5A). The most up-regulated genes were found in 1-year-old taproots, and the genes encoding peroxidase (PER), caffeoyl-CoA O-methyltransferase (CCoAOMT), and 4-coumarin: CoA ligase (4CL) were highly expressed. The number of up-regulated genes in 2-year-old taproots decreased gradually, although the genes encoding cinnamyl alcohol dehydrogenase (CAD) and cinnamoyl-CoA reductase (CCR) were significantly up-regulated; The number of up-regulated genes in 3-year-old taproots was the lowest of all the test years, and genes encoding beta-glucosidase and peroxidase were significantly up-regulated (Fig. 5B; Table S10).

### Differential expression of genes related to the triterpene saponins biosynthesis pathway

**Fig. 6:**
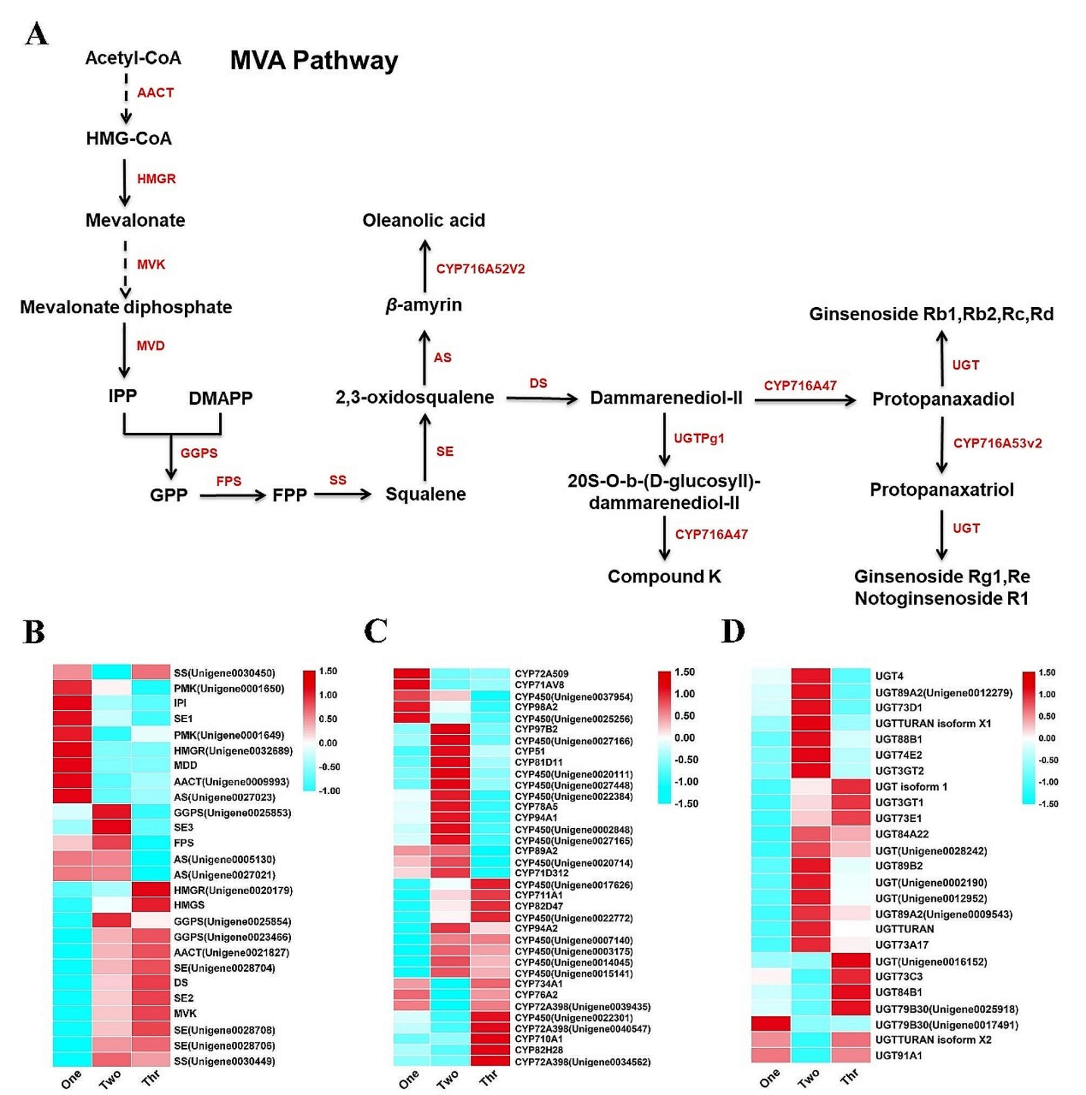
Significant enrichment pathway and DEGs related to triterpene saponin biosynthesis. (**A**) Partial pathway of triterpene saponin biosynthesis. (**B**) Heatmap of genes involved in triterpene saponin precursors. (**C**) Heatmap of CYPs involved in triterpene saponin monomers. (**D**) Heatmap of UGTs involved in triterpene saponin monomers

Assessment of saponin content and DAMs analysis showed that levels of triterpenoid saponins in *P. notoginseng* taproots increased with plant age over the first three years (Fig. S1 and S3). Mevalonic acid (MVA) pathway is the main biosynthesis pathway for triterpenoid saponins. Synthesized isopentenyl pyrophosphate (IPP) and dimethylallyl pyrophosphate (DMPP) undergo a catalyzed reaction to synthesize 2,3-oxidosaqualene, which is then cyclized, hydroxylated and glycosylated successively to finally form triterpenoid saponins (Fig. 6A) [22]. The heatmap of DEGs from this study showed that upstream genes involved in triterpenoid saponins synthesis, including 3-hydroxy-3-methylglutaryl coenzyme A reductase (HMGR), mevalonate diphosphate decarboxylase (MDD), isopentenyl diphosphate isomerase (IPI), acetyl-CoA C-acetyltransferase (AACT), and β-amyrin synthase (AS) were up-regulated differently in 1-, 2- and 3-year-old taproots (Fig. 6B; Table S11), while CYP450s and UGTs involved in the downstream catalysis of triterpenoid saponins were up-regulated most in 2- and 3-year-old taproots (Fig. 6C and D; Table S11).

### Integrated analysis of DAMs and DEGs

**Fig. 7:**
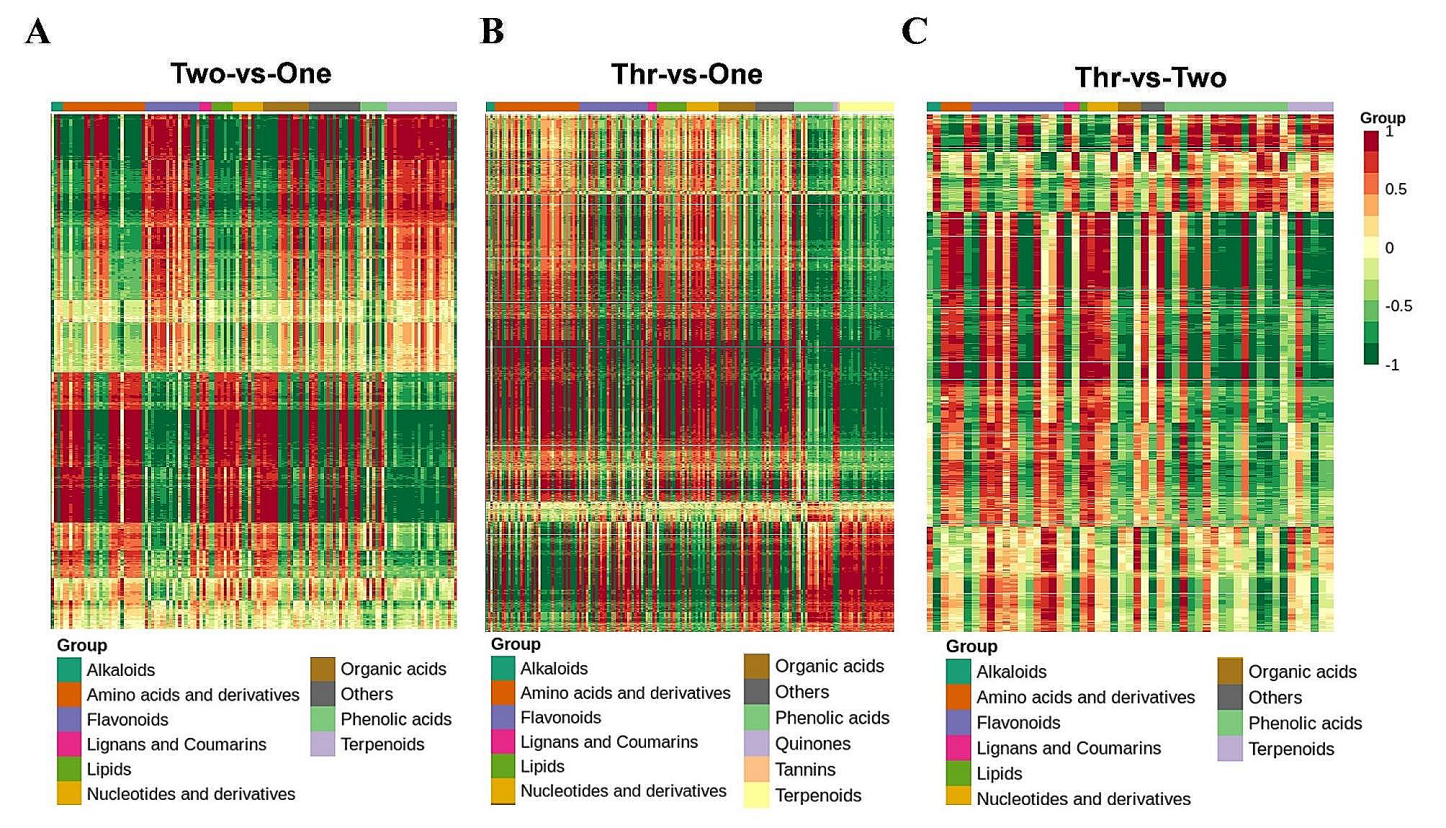
Heatmap correlation analysis of DEGs and DAMs in all comparison groups. Red represents a positive correlation and green represents a negative correlation

To understand the regulatory networks of dynamic metabolic processes of *P. notoginseng* taproots in three growth years, we tried to integrate the metabolome and transcriptome data by correlation analysis. The KEGG enrichment analysis of DAMs and DEGs showed that the DEGs were significantly enriched in multiple pathways (*p* value < 0.01), while the DAMs were only enriched in the phenylpropanoid biosynthesis pathway (*p* value < 0.01) (Fig. S6), suggesting that the phenylpropanoid biosynthesis pathway plays an important role in the development of and metabolic changes observed in *P. notoginseng* taproots. The correlation analysis of DAMs and DEGs (Correlation coefficient > 0.8) found that amino acids and their derivatives, flavonoids and terpenoids had the highest correlation in the Two-vs-One comparison group (Fig. 7A); Amino acids and derivatives, flavonoids, and terpenoids had the highest correlation in the Thr-vs-One comparison group (Fig. 7B), and phenolic acids, flavonoids, and terpenoids had the highest correlation in the Thr-vs-Two comparison group (Fig. 7C). These results supports those from the metabolomic analysis.

**Fig. 8:**
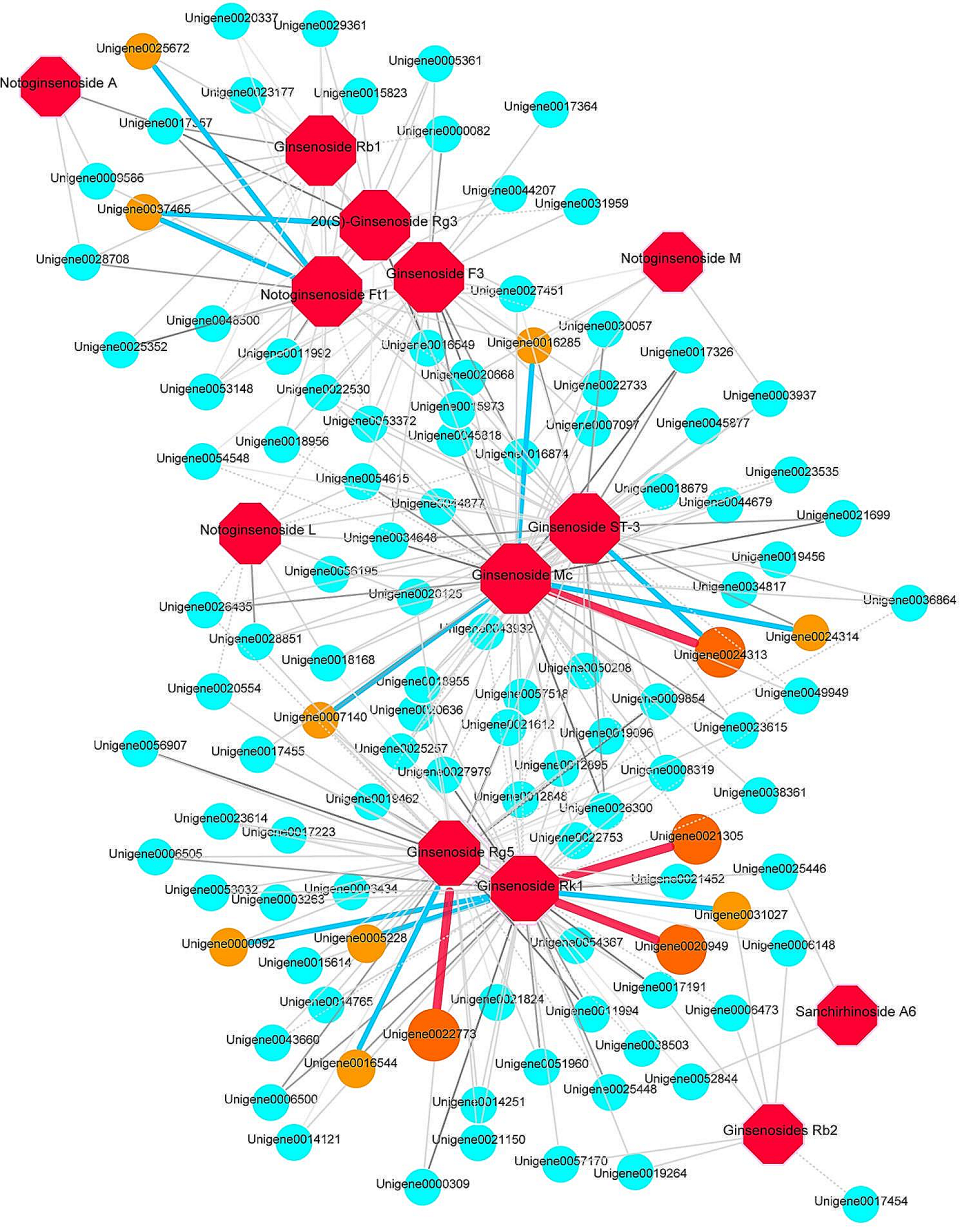
Regulatory network of DEGs highly related to triterpene saponins biosynthesis. The red octagons represent triterpenoid saponins. Orange-red circles represent genes associated with a correlation coefficient of 0.999. Orange-yellow circles represent genes associated with a correlation coefficient of 0.998

Further enrichment and correlation analysis of DAMs and DEGs highly related to triterpenoid saponins synthesis (Pearson correlation coefficient > 0.99) showed that 414 genes were highly correlated with different triterpenoid saponins, and that the number of genes associated with ginsenoside RK1, Rg5, Mc and ST-3 was the largest (Table S12). By constructing regulatory networks of genes highly related to at least two triterpenoid saponins, it was found that four genes encoding 2-nonaprenyl-3-methyl-6-methoxy-1,4-benzoquinol hydroxylase, isoflavone 3’-hydroxylase-like isoform X2, RNase-like major storage protein, and tetratricopeptide repeat (TPR)-like superfamily protein were highly related to ginsenoside Rg5, ginsenoside Rk1, and ginsenoside Mc (PCC > 0.999), while 9 genes encoding CYP450, transcriptional regulator EFH1 isoform X2, glucan endo-1,3-beta-glucosidase-like isoform X1, RNase-like major storage protein, sterol 4-C-methyltransferase strm-1-like, tetratricopeptide-like helical, UGT14, vacuolar-processing enzyme isoform X2, and DUF581 domain-containing protein were highly associated with notoginsenoside Ft1, 20(*S*)-ginsenoside Rg3, ginsenoside Mc, ginsenoside Rk1, and ginsenoside Rg5 (Fig. 8; Table S13).

## Discussion

The accumulation of metabolites in plants is characterized by spatiotemporal changes, and is closely related to plant development and reaction to changes in the its surrounding environments. Primary metabolites, including carbohydrates, lipids, nucleic acids and proteins, are essential for life processes, and mainly provide material and energy for cell morphogenesis and physiological processes [1–4, 23]. In contrast, secondary metabolites are synthesized on the basis of primary metabolites, and are not necessary for plant growth and development, but rather play an important role in the interaction between plants and their environments [1, 7]. Here, investigation of the metabolome showed that the distribution of DAMs in *P. notoginseng* taproots was significantly different in different growth years, and that primary metabolites such as amino acids and derivatives, lipids, nucleotides and derivatives, accumulated mainly in the 1-year-old taproots (Fig. 2B and Table S4). This is consistent with similar results from potato. During the development of potato tubers, the concentrations of sucrose, glucose and fructose in immature tubers were the highest, and the expression levels of arginine decarboxylase and ornithine decarboxylase were also high [24], indicating that the development of young tissues is related to vigorous primary metabolism. Furthermore, in *Arabidopsis*, the decrease of histidine in histidinol-phosphate aminotransferase 1 (*hpa1*) mutant led to shortening of the root, while the decrease of serine in a *gapcp* double mutant resulted in defective primary root growth, indicating that amino acids are necessary for the maintenance of carbohydrate biosynthesis and for the normal growth of roots [25, 26]. Our previous studies confirmed that the accumulation of arginine plays an important role in regulating the growth and development of 1-year-old *P. notoginseng* taproots [19]. In rice, mutation of *OsASL*, which encodes argininosuccinate lyase involved in arginine synthesis, resulted in a short-root phenotype, while exogenous arginine was able to restore the normal growth of roots, showing that arginine promotes the root elongation of rice by affecting root cell division [27]. Moreover, certain amino acids are also precursors of secondary metabolites, such as acetyl coenzyme A, which are formed by the degradation of leucine, isoleucine, lysine and valine, and are important precursor for the synthesis of terpenoids [14, 22, 28–31]. Additionally, many key physiological processes in plants, such as water regulation, cytoskeleton activity, programmed cell death (PCD) activation or root hair growth, are regulated by complex signal transduction networks mediated by lipids [32–36]. Thus, we hypothesized that the accumulation of a large number of primary metabolites is necessary for the rapid development of the young tissues in 1-year-old *P. notoginseng* taproots, and that these primary metabolites also provides precursors for secondary metabolites production at the later stages of development.

The metabolome analysis demonstrated that there was a significant accumulation of flavonoids and terpenoids were significantly accumulated in 2- and 3-year-old *P. notoginseng* taproots, while phenolic acids predominantly accumulated in 3-year-old *P. notoginseng* taproots (Fig. 2B and Table S4). This result was supported by the transcriptome and metabolome correlation analysis (Fig. 7). Previous studies have demonstrated that levels of triterpenoid saponins increase with the age of *P. notoginseng* taproots, which is an important basis for the quality and determination of the harvest time of *P. notoginseng* [17, 18, 20]. Recent studies showed that the release of triterpenoid saponins could induce degradation of root cell walls and aggravate root rot by modifying the rhizospheric microbiome surrounding *P. notoginseng* roots [37, 38]. Other studies have shown that the secretion of phenolic acids is able to influence the soil microbial flora by stimulating soil-borne pathogens and inhibiting beneficial microorganisms, ultimately facilitating the occurrence of root rot in *P. notoginseng* [39–42]. Therefore, triterpenoid saponins and phenolic acids secreted by *P. notoginseng* into the rhizosphere can mediate changes in the structure and functional diversity of the soil microbial community, which may be closely related to the accumulation of triterpenoid saponins and phenolic acids in mature *P. notoginseng* roots.

In the transcriptome analysis, DEGs involved in phenylpropanoid biosynthesis, metabolic pathway and biosynthesis of secondary metabolites were found to be significantly enriched in 1-, 2- and 3-year-old *P. notoginseng* taproots (Fig. 3D). In a previous study, we also found that DEGs associated with plant hormone signal transduction, starch and sucrose metabolism, and phenylpropanoid biosynthesis were closely related to the taproot thickening in 1-year-old *P. notoginseng* [19]. These results indicate that starch, sucrose, plant hormones as well as the above-mentioned primary metabolites may participate in the development of the tissues of young taproots. Notably, DEGs involved in phenylpropanoid biosynthesis were identified as being significantly enriched in combined transcriptome and metabolome analyses in the study (Fig. S6). Phenylpropanoids participate in the synthesis of many secondary metabolites through the action of certain superfamilies of enzymes, including oxidases, ligases, oxidoreductases, and transferases [21, 43–45]. In potato, phenylpropanoid metabolism changes significantly with the growing season and is closely related to development and quality [24]. Here, DEGs involved in phenylpropanoid biosynthesis pathway were identified in *P. notoginseng* taproots from different growth years, and were mainly involved in the synthesis of P-hydroxy-phenyl lignin, guaiacyl lignin, 5-hydroxyguaiacyl lignin and syringal lignin (Fig. 5). Lignin is crucial to the integrity, hardness and strength of plant cell wall, and plays roles in the cell wall extensibility and cell expansion, the vascular system transport of water and solute, and the defense against pathogenic organisms [46–48]. Therefore, we speculate that a large number of DEGs and DAMs associated with flavonoids and phenolic acids identified in 2- and 3-year-old *P. notoginseng* taproots may be related to the phenylpropanoid biosynthesis pathway. Our results also suggest that the phenylpropanoid biosynthesis pathway played dual roles both in taproot thickening and synthesis of secondary metabolites in *P. notoginseng*.

High levels of triterpenoid saponins accumulation are a primary goal of *P. notoginseng* breeding. In this study, high levels of the triterpenoid saponins were mainly found in 2- and 3-year-old *P. notoginseng* taproots (Fig. S3). Moreover, downstream genes of the CYP450s and UGTs involved in the biosynthesis of triterpenoid saponins had same expression patterns (Fig. 6C and D). Previous studies have found genes encoding farnesyl diphosphate synthesis (FPS), squalene epoxidase (SE), and damarenediol-II synthesis (DS) were more highly expressed in the shoots than that in the roots [17, 20], while CYP450s is highly expressed in the roots [49], speculating that triterpenoid saponins may be synthesized in shoots and then transferred to the roots. In this study, 16 triterpenoid saponins were found to be highly correlated with multiple genes involved in triterpenoid saponins synthesis, including those encoding SE, DS, UGTs and CYP450s, which are highly correlated with the synthesis of ginsenoside Rg3 (Fig. 8; Table S13). This suggests that certain triterpenoid saponins may be synthesized directly in the roots, or perhaps that precursors are transferred, but subsequent synthesis of monomer saponins still depends on the synthase genes highly expressed in the roots. The gene encoding RNase-like major storage protein was identified as being important for taproot thickening in 1-year-old *P. notoginseng* [19]. Interestingly, we also found that this gene was highly associated with multiple triterpenoid saponins. So, this gene may play a dual role both in development and quality of *P. notoginseng.* All of the above results help to further clarify a basis for the development of quality in *P. notoginseng*.

In this study, we systematically analyzed the dynamic changes occurring in different metabolites during the development of *P. notoginseng* taproots, and further clarified the distribution of various metabolites at different growth stages. We found that amino acids and related genes were mainly upregulated in 1-year-old taproots, while flavonoids, terpenoids and related genes were predominantly upregulated in 2-, 3-year-old roots. Meanwhile, this study has identified some key candidate genes related to saponin synthesis and quality breeding. These results will contribute to scientific guidance for the field management of *P. notoginseng* in the future.

## Conclusions

This study analyzed the significantly DAMs in *P. notoginseng* taproots over the first three growth years, and identified some CYP450 and UGTs genes closely related to the accumulation of triterpenoid saponins. These results provide a theoretical basis for the planting and harvesting of *P. notoginseng*.

## Materials and methods

### Plant materials

Our previous study investigating the dynamics of growth and accumulation of saponins in 1-, 2- and 3-year-old *P. notoginseng* taproots showed that July was a critical period for rapid root expansion and that significant accumulation of saponins occurred at this time. Therefore, in this study, we collected our plant samples in July. 1-, 2- and 3-year-old *P. notoginseng* plants were selected from Wenshan Miaoxiang *P. notoginseng* Industrial Co., Ltd, at their Xiaoxinzhai base (104°24’29.0484"E, 23°24’8.496"N, Altitude: 1490 m) (Fig. 1A). Official permits were not required for the collection of *P. notoginseng*, because the species is not a national key protected plant, and because the specimens were not taken from wild populations. The formal identification of morphological characters was performed by Professor Shengchao Yang of Yunnan Agricultural University, and a *P. notoginseng* voucher specimen (voucher # KUN 1,372,042) was deposited in herbarium of the Kunming Institute of Botany, the Chinese Academy of Sciences. After washing with clean water, the taproots were removed and cut into 0.5 cm pieces, then stored in liquid nitrogen in 25 mL centrifuge tubes.

### Morphological statistics

The taproots were cut into two parts from the basal part of the stem. The length of each taproot was measured with a ruler, and vernier calipers were used to determine its maximum diameter. The fresh taproots were weighed on an electronic balance, and were then dried to constant weight at 60 °C in an oven. Thirty replicates per test group were collected. Statistical analysis was conducted in SPASS.

### Paraffin section

For examination of anatomical structure, 2 mm slices were cut from the fresh taproots, and were immediately submersed in 5 mL of a solution of formalin-glacial acetic acid-alcohol (FAA) and left for 48 h. Preparation of paraffin sections followed the method described in Canene-Adams [50].

### Determination of total saponins

Fresh taproots were collected from 1-, 2- and 3-year-old plants in July. Taproots were then washed with clean water and were dried to constant weight in an oven at 60 °C. The dried material was ground by passing it through a sieve (80-mesh), and 0.6 g of the resulting powder was shaken with 50 mL 70% methanol and left to stand for half an hour, after which it was ultrasonicated for 30 min. A microporous filter (0.22 μm) was used to filter the test solution, and the filtered samples were then subjected to high-performance liquid chromatography (HPLC) to determine the total saponin content. An injection volume of 10 µL was used, and chromatographic conditions were as follows: column (250 × 4.6 mm, 5 μm), and the column temperature was maintained at 25 °C, the mobile phase comprised water (A)- and acetonitrile (B), detection wavelength was 203 nm, and the flow rate was 1.5 mL/min.

### Widely targeted metabolome analysis

A vacuum freeze-drying machine (Scientz-100 F) was used to dry the samples, and they were then ground to powder using an MM 400 (Retsch). Samples were then subjected to methanol extraction. 100 mg powder was dissolved in 0.6 mL methanol (70%), and stored overnight at 4 °C in a refrigerator. Samples were swirled six times during this time to improve the efficiency of the extraction. The following day, the solution was centrifuged for 10 min, and the supernatant was extracted and filtered in preparation for UPLC-MS/MS analysis.

The metabolome analysis was conducted by the Metware Biotechnology Co., Ltd. (Wuhan, China). After metabolites from different samples had been tested, all mass spectrometry peaks were analyzed using peak area integration, with integration correction being performed on the peaks resulting from the same metabolite but from different samples. DAMs were identified as those with fold change ≥ 2 or ≤ 0.5 and having a variable importance in the project ≥ 1. KEGG enrichment analysis was then performed on the DAMs.

### Transcriptome sequencing analysis

Total RNA was extracted from the samples, and to construct sequencing libraries. The libraries were sequenced using an Illumina HiSeqTM4000. The reads were filtered for quality and trimmed, and then assembled reads [51]. The unigenes were functionally-annotated by searching against major databases, including GO, Pfam, KOG, KO, Swiss-Prot, and NR using BLAST (*E* < 10^− 5^). Differentially expressed genes (DEGs) were then identified as those with FDR < 0.05 and | log_2_FC | > 1. Gene ontology functional enrichment and pathway significant enrichment analyses were then performed, with significantly enriched pathways being defined as those with a Q-value ≤ 0.05. The expression levels of unigenes were calculated and then were normalized using RPKM (Reads Per kb per Million reads) [52]. A principal component analysis of the samples was performed in R version 1.6.2. Heatmaps were generated in TBtools to allow hierarchical clustering analysis of gene expression across different samples. Raw data generated in this study were submitted to the NCBI Sequence Read Archive database, under the accession number PRJNA881712.

### RT-qPCR analysis

The RNA-seq results were then validated. Total RNA from *P. notoginseng* taproots from 1-, 2-, and 3-year-old plants was extracted using a kit (Magen Biotech Co., Ltd., China). A second kit (Tsingke Biotech Co., Ltd., China) was used to reverse transcribed the RNA to cDNA. An ABI QuantStudio 5 Real-Time PCR System (Applied Biosystems) was used to perform the RT-qPCR reactions. The primers used in the reactions were designed using Primer 3 web version 4.1.0, and are given in Table S1. The *YLS8* gene was used as an endogenous control, and relative gene expression was calculated using the 2^−ΔΔCT^ method.

### Correlation analysis

A correlation analysis between the transcriptome and metabolome was performed, 110 genes highly correlated with at least two saponins were screened according their Pearson correlation coefficient (PCC > 0.99), and a regulatory network diagram was drawn using the online platform from Gene Denovo Biotechnology Inc (Guangzhou, China).

### Statistical analysis

Samples were analyzed statistically using a one-way analysis of variance at the *P* ≤ 0.05 level.

### Electronic supplementary material

Below is the link to the electronic supplementary material.

Supplementary Material 1

Supplementary Material 2

## Data Availability

The original contributions presented in the study are publicly available. The data have been deposited in the NCBI Sequence Read Archive database under accession number: PRJNA881712.

## Abbreviations

4CL: 4-coumarin: CoA ligase
AACT: Acetyl-CoA C-acetyltransferase
AS: β-amyrin synthase
CAD: Cinnamyl alcohol dehydrogenase
CCR: Cinnamoyl-CoA reductase
CCoAOMT: Caffeoyl-CoA O-methyltransferase
CYP450s: Cytochrome P450s
DMPP: Dimethylallyl pyrophosphate
DS: Damarenediol-II synthesis
FAA: Formalin-glacial acetic acid-alcohol
FPS: Farnesyl diphosphate synthesis
HMGR: 3-hydroxy-3-methylglutaryl coenzyme A reductase
HPLC: High-performance liquid chromatography
IPI: Isopentenyl diphosphate isomerase
IPP: Isopentenyl pyrophosphate
LC − MS/MS: Liquid chromatography − tandem mass spectrometry
MDD: Mevalonate diphosphate decarboxylase
OPLS-DA: Orthogonal partial least-squares discriminant analysis
PCA: Principal component analysis
PCD: Programmed cell death
PER: Peroxidase
RPKM: Reads per kb per million reads
SE: Squalene epoxidase
UGTs: Uridine diphosphate-dependent glycosyltransferases
