# Supplementary material for: Integrated metabolome and transcriptome analyses reveal the molecular mechanism underlying dynamic metabolic processes during taproot development of Panax notoginseng
Source: BMC Plant Biol. 2024 Mar 5;24:170. doi: 10.1186/s12870-024-04861-8 (PMC10913227; doi:10.1186/s12870-024-04861-8)
Supplement: Supplementary file 1 — Supplementary Material 1 [file 12870_2024_4861_MOESM1_ESM.docx]

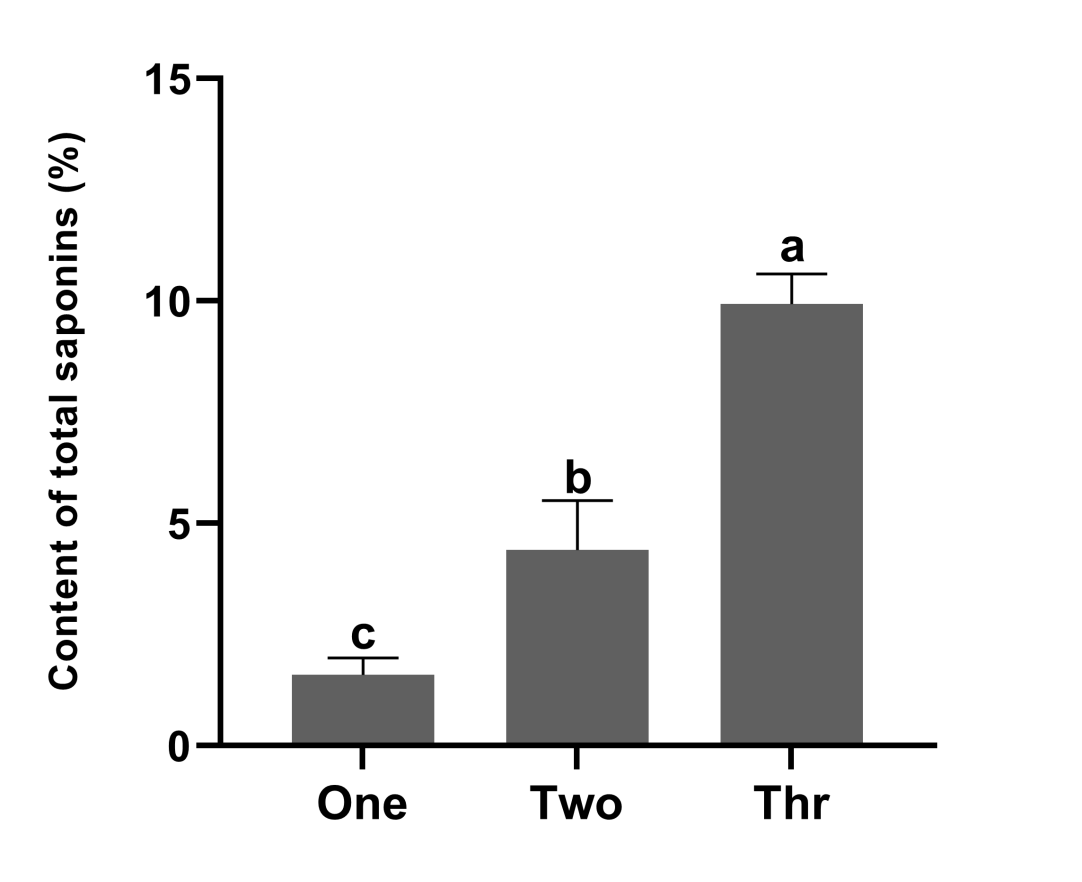


**Fig. S1.** Total saponins content of *P.notoginseng* taproots in three growing year**s.**


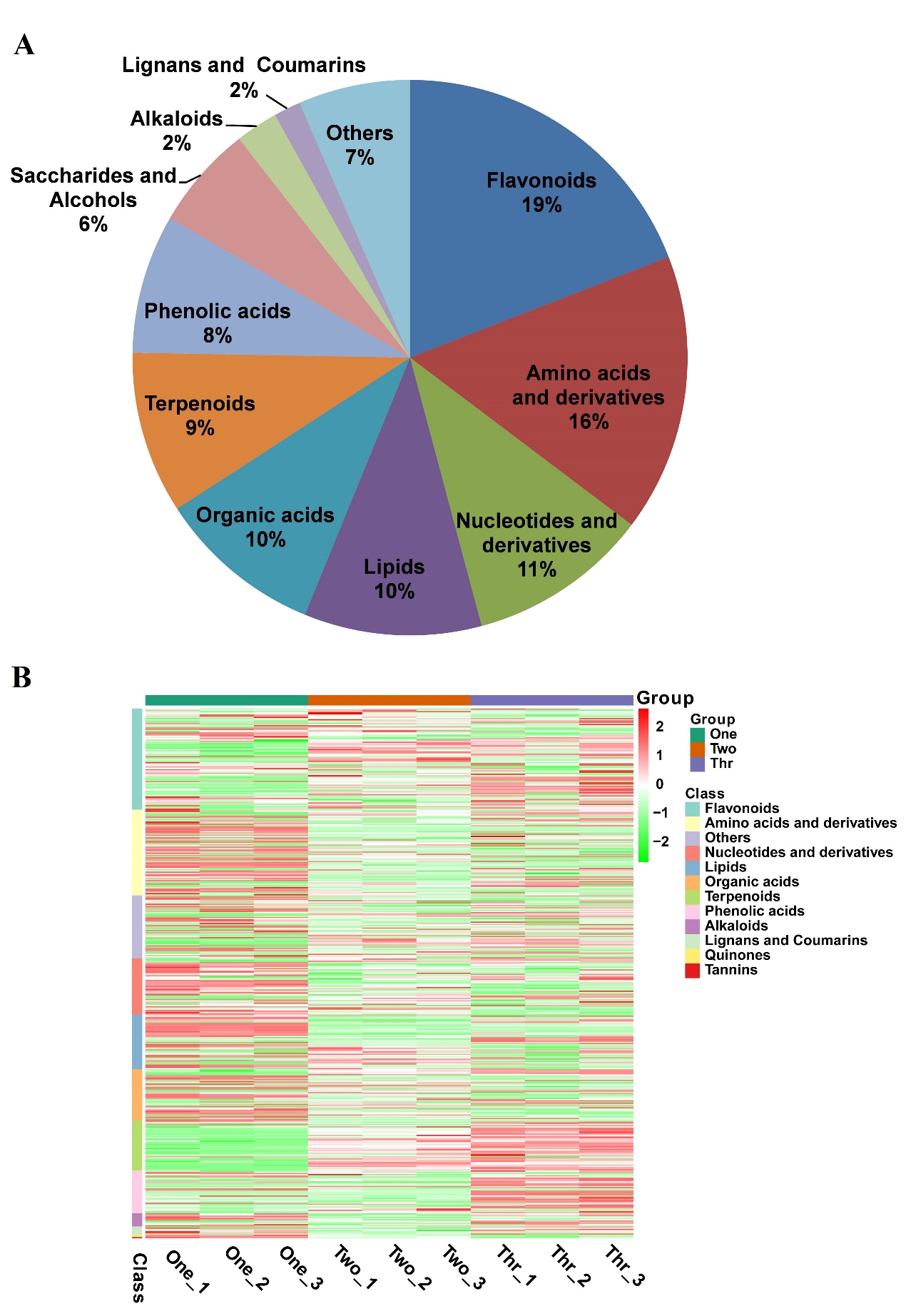


**Fig. S2.** Metabolites classification and expression of *P. notoginseng* taproots in three growing years. (A) Cluster analysis. (B) Expression heatmap of metabolites, the color indicates the relative concentration of metabolites from low (green) to high (red).


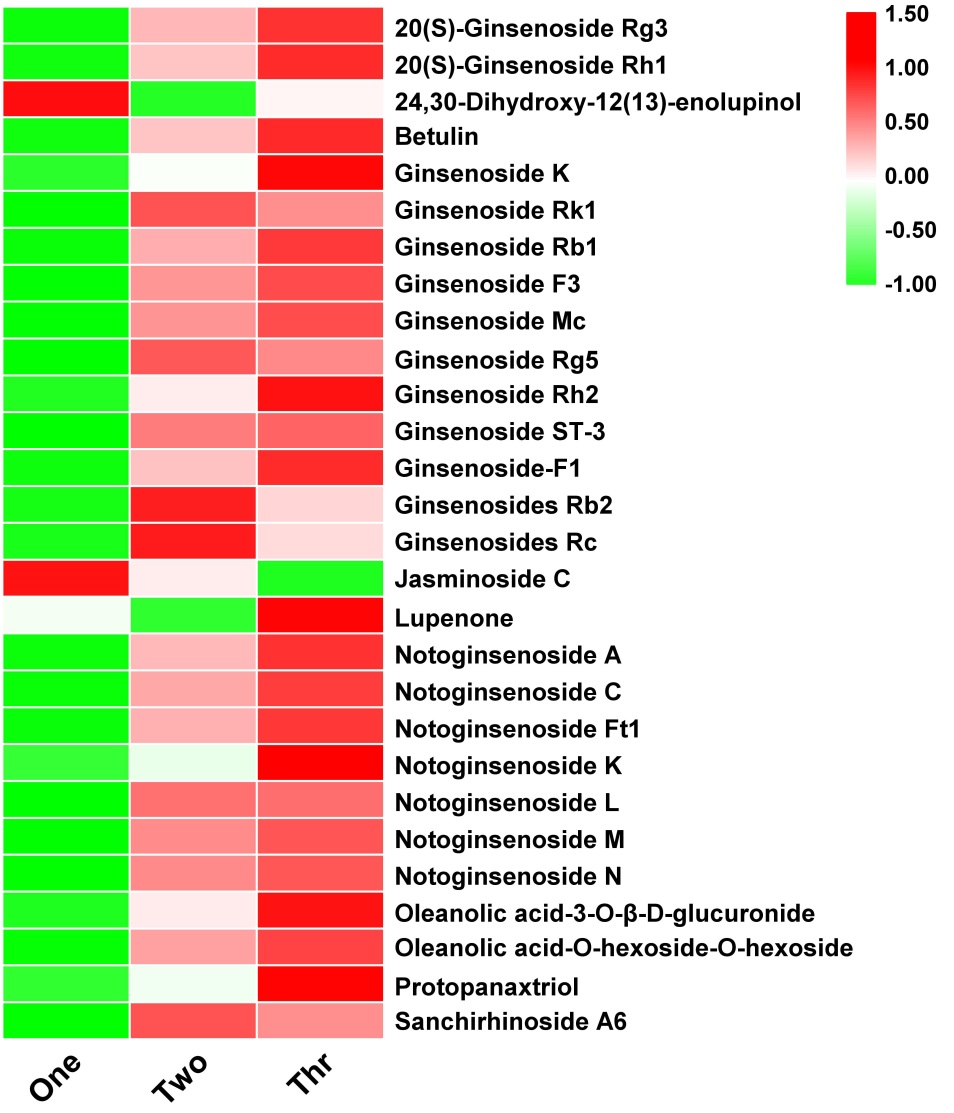


**Fig. S3.** Heatmap of terpenoids content in *P. notoginseng* taproots with three growing years.


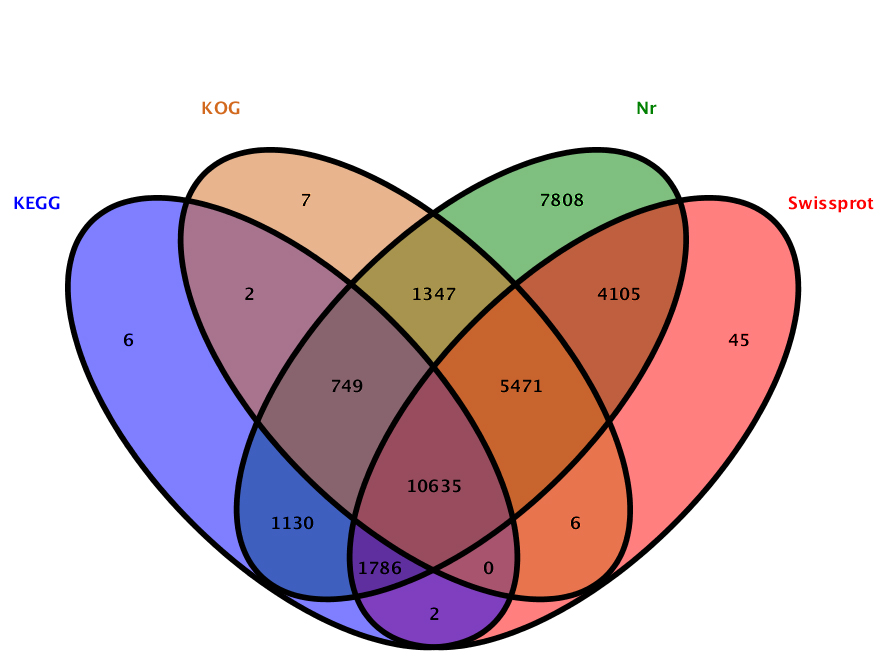


**Fig. S4.** Venn diagram of annotation results in four database.


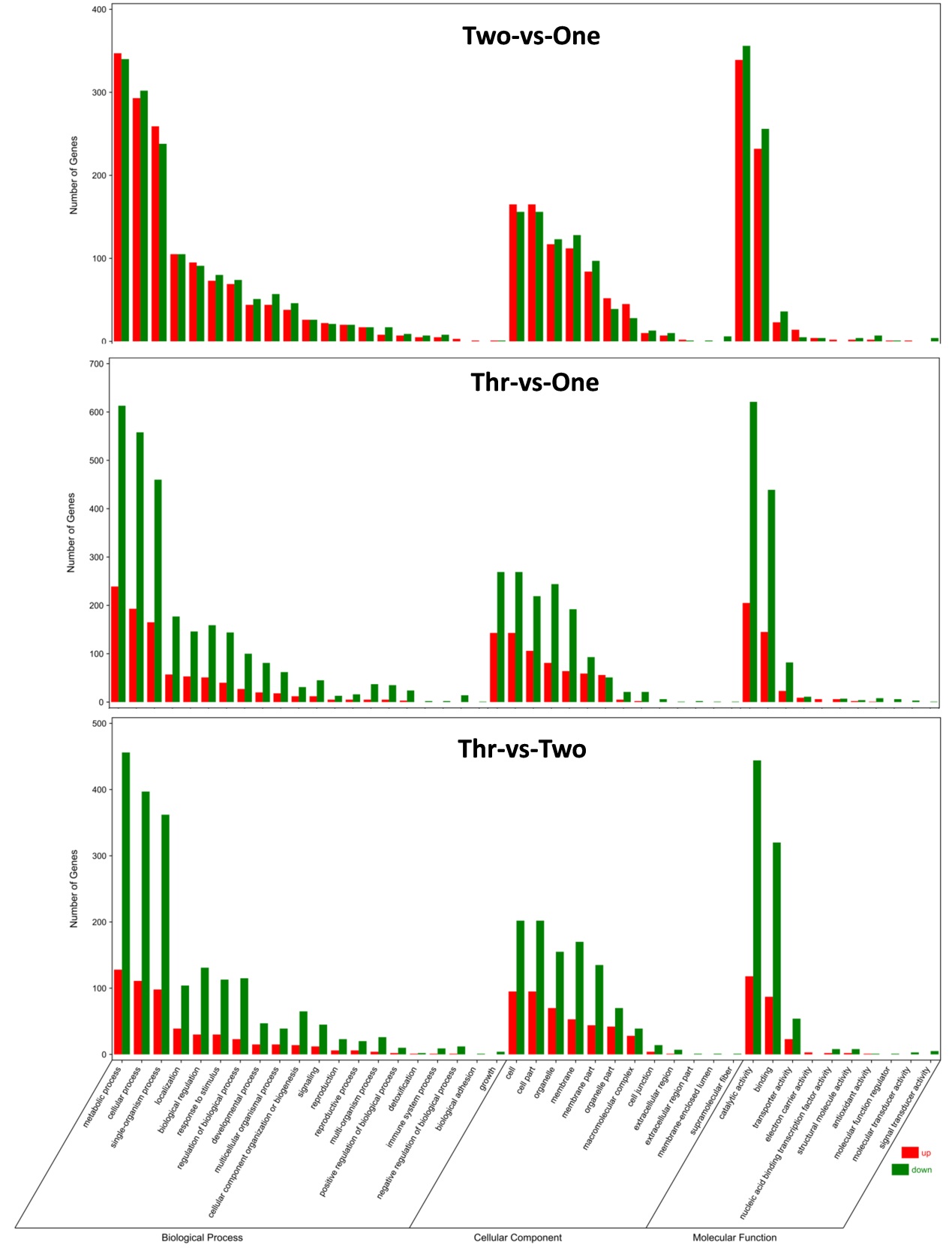


**Fig. S5.** GO enrichment analysis of DEGs in all comparison groups.


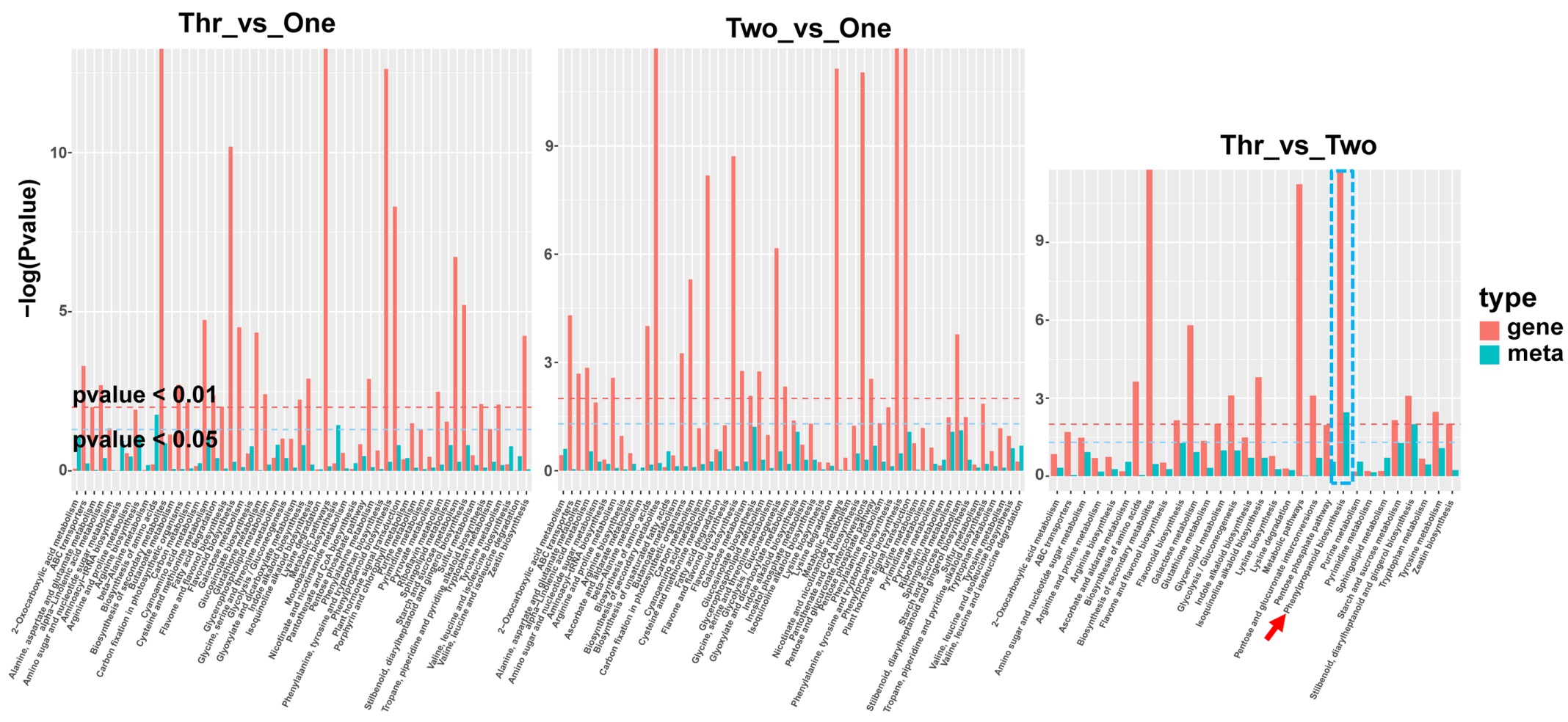


**Fig. S6.** KEGG enrichment analysis of DEGs and DAMs in all comparison groups.The vertical axis represents the enriched KEGG pathways. The horizontal axis represents the number of DEGs (red) and DAMs (blue) in each pathway.
